# Well-being and *Buen Vivir*: intercultural dialogue between *etic* and *emic* perspectives

**DOI:** 10.3389/fpsyg.2026.1938770

**Published:** 2026-10-01

**Authors:** Daniela S. Zanini, Iorhana Fernandes, Tamiris M. Mendonça, Elias Nazareno

**Affiliations:** 1 Pontifícia Universidade Católica de Goiás, Goiânia, Brazil; 2 Universidade Federal de Goias, Goiânia, Brazil

**Keywords:** decolonial, emic perspective, etic perspective, indigenous, well-being

## Abstract

This study aimed to analyze perceptions of well-being, happiness, and meaning in life among non-indigenous and indigenous participants from the Javaé and Karajá peoples, based on *etic* and *emic* perspectives. A qualitative, exploratory, and comparative design was adopted. Participants included 13 indigenous students enrolled in the intercultural education degree program at the Federal University of Goiás and 14 non-indigenous individuals living in the central-west region of Brazil. Data were collected through three open-ended questions addressing well-being, happiness, and meaning in life. Interviews were transcribed verbatim and analyzed using initial and focused coding procedures grounded in constructivist grounded theory, supported by computational tools based on large language models. The findings revealed common categories across both groups, such as family and community relationships, meaningful work and teaching, physical and mental health, and realization of aspirations. However, these categories acquire distinct meanings across different cultural frameworks. Among indigenous participants, well-being was conceived as a relational, collective, and territorially grounded experience, integrating culture, spirituality, ancestry, and nature. In contrast, non-indigenous participants emphasized more individualized narratives, centered on personal progress, consumption, and immediate pleasures. These results highlight the importance of intercultural and decolonial approaches to psychological research on well-being and challenge the universalization of models derived predominantly from WEIRD populations.

## 1 Introduction

The study of well-being occupies a central position in contemporary psychological sciences. In recent decades, this field has been marked by critiques of the universality of traditional measures and by calls for approaches that are sensitive to cultural, social, and contextual dimensions (Lomas et al., 2021). This movement stems from the recognition that a substantial proportion of the available theories and instruments have been developed based on WEIRD samples (Western, Educated, Industrialized, Rich, and Democratic), which limits their external validity and their ability to capture the diversity of human experiences (Henrich et al., 2010).

Well-being has been conceptualized in psychological research as a multidimensional construct encompassing different aspects of positive functioning and experience. Influential approaches distinguish hedonic dimensions, related to positive affect and evaluations of life, from eudaimonic dimensions, associated with meaning, purpose, positive relationships, and personal development (Ryff, 2022; Seligman, 2011, 2018). At the same time, there is no full consensus regarding the constitutive dimensions or conceptual boundaries of well-being, and considerable overlap exists with related phenomena such as happiness and meaning in life (Pincus, 2024; Zhang et al., 2024). In the present study, this multidimensional psychological understanding of well-being is adopted as an initial theoretical framework rather than as a culturally universal definition. We recognize that this framework has been developed predominantly within Western psychological traditions and therefore constitutes an etic starting point for the investigation. Accordingly, happiness, positive evaluations of life, and meaning or purpose provide broad domains through which participants were invited to articulate what contributes to living and feeling well, while the meanings attributed to these domains were examined as culturally situated rather than assumed to be equivalent across groups.

Several theoretical models have sought to organize these dimensions of well-being, including subjective well-being (Diener, 1984), psychological well-being (Ryff, 1989), self-determination theory (Deci and Ryan, 2002), and integrative frameworks such as PERMA (Seligman, 2011), although relevant, tend to reflect individualistic assumptions that are often disconnected from social inequalities and cultural contexts (Lomas et al., 2021). Cross-cultural research indicates that definitions of well-being vary according to historical, cultural, and economic contexts (Sollis et al., 2024). Interdependent conceptions, centered on belonging, social bonds, and social harmony, demonstrate greater cross-cultural stability, whereas independent conceptions, oriented toward autonomy and self-expression, predominate in WEIRD societies (Gardiner et al., 2020).

In this context, the concepts of etic and emic offer a relevant theoretical and methodological framework for examining how well-being is understood across cultures. The etic perspective approaches a phenomenon through concepts and interpretive frameworks that are external to a particular cultural system, whereas the emic perspective seeks to understand meanings, values, and practices from within the cultural framework in which they are produced (Helfrich, 1999; Sollis et al., 2024). Thus, the distinction between etic and emic does not depend on whether a particular theme is shared across groups or appears only in one of them. Similar themes may acquire different culturally situated meanings, just as apparently group-specific elements may still be interpreted through externally defined categories. Rather than representing opposing approaches, etic and emic perspectives can be used complementarily to examine the relationships between externally informed analytical frameworks and culturally situated systems of meaning.

Importantly, this framework also encourages critical reflection on the cultural and moral assumptions embedded in dominant psychological theories. As Christopher and Hickinbottom (2008) contend, positive psychology has often treated values associated with western individualism as though they were culturally neutral and universally applicable. Such assumptions may obscure alternative conceptions of well-being grounded in collective identity, reciprocity, spirituality, and relationships with community and territory, thereby reinforcing the underrepresentation of non-Western epistemologies in psychological science.

Within Latin American debates, Indigenous epistemologies have contributed significantly to alternative understandings of living well, including perspectives associated with *Buen Vivir*, which conceive well-being as inseparable from collectivity, spirituality, relationships with nature, and intergenerational responsibility. In contrast to dominant conceptions of well-being, which have largely emphasized individual self-realization, Indigenous perspectives are grounded in relational value systems that challenge the underlying assumptions of Western psychology (Prilleltensky, 2013) and offer alternative ways of understanding what it means to live well (Acosta, 2016; Zanini et al., 2025). Bringing these perspectives into dialogue not only broadens contemporary understandings of well-being but also fosters a more equitable intercultural psychology by recognizing epistemic plurality and advancing epistemic justice (Prilleltensky, 2013).

Despite the relevance of these issues, empirical studies examining how dimensions associated with well-being are understood and articulated across culturally distinct groups, such as Indigenous and non-Indigenous populations, remain scarce. In light of this gap, the present study aims to analyze convergences and divergences in how Javaé and Karajá participants from the Central-West region of Brazil and non-Indigenous participants understand and articulate experiences associated with well-being, particularly happiness, feeling well about oneself and one’s life, and meaning in life, through an etic–emic framework. This investigation is guided by the following central research question: What convergences and divergences characterize the ways Indigenous and non-Indigenous participants understand and articulate these dimensions of well-being? By addressing this question, the study seeks to contribute to a more plural and culturally situated Psychology, committed to epistemic justice and to the recognition of diverse ways of living and understanding the world. More specifically, it seeks to explore the concept of well-being through a perspective that is more representative of human diversity, rather than being limited to Western societies and WEIRD populations or sample.

## 2 Methods

### 2.1 Participants

The Indigenous group consisted of four women from the Javaé people, aged between 24 and 28 years (M = 25.25; SD = 2.75), residing in the villages of Txuiri, Imoti II, Wari-wari, and Canoanã, located on Bananal Island, in the state of Tocantins (TO), and nine Indigenous men from the Javaé and Karajá peoples, aged between 24 and 38 years (M = 33.88; SD = 5.30). The Javaé participants came from the villages of Txuiri, Canoanã, Taimy, and Boa Esperança (TO) whereas the Karajá participants resided in Hawalorá, Macaúba, Ibutuna, and Xexotãwa (state of Mato Grosso—MT). All Indigenous participants were students in the Intercultural Education degree program at the Federal University of Goiás (UFG).

The non-Indigenous group consisted of six women aged between 22 and 28 years (M = 23.16; SD = 3.31), all of whom identified as White, cisgender, and heterosexual, with either incomplete (*n* = 4) or completed (*n* = 2) higher education. The male participants included eight individuals aged between 22 and 44 years (M = 30.25; SD = 6.90), who identified as White (*n* = 4) or Brown (*n* = 5), cisgender, and heterosexual, with either incomplete (*n* = 2) or completed (*n* = 6) higher education. All participants resided in the Central-West region of Brazil.

Participants were selected through convenience sampling. Inclusion criteria were being over 18 years of age, agreeing to participate in the study by signing an informed consent form, and responding to the three guiding questions. No exclusion criteria were established.

### 2.2 Cultural context of the indigenous peoples

The Javaé and Karajá peoples belong to the Macro-Jê linguistic stock and the Karajá language family and have lived in the Central-West region of Brazil for thousands of years (Pereira and Nazareno, 2021). The two Indigenous peoples, *Bero Biawa Mahãdu* (“People of the River”), the Javaé, and *Itya Mahãdu* (“People of the Middle”), the Karajá, inhabit Bananal Island, located in the states of Tocantins and Mato Grosso, along the banks of the Araguaia and Javaés rivers (Rodrigues, 2008). Both names are self-designations in their Indigenous language, *Iny Rybè* (Iny Language). Their current populations are approximately 1,700 and 4,000 individuals for the Javaé and Karajá peoples, respectively.

Both peoples have cosmologies that are partly grounded in matrilineality. In other words, kinship and descent are organized according to the ancestry of the mother and her blood relatives. According to Rodrigues (2008), among the Javaé and Karajá peoples, marriage gives rise to uxorilocal residence patterns. This means that, following marriage, men are expected to move to their wives’ households and assume responsibility for supporting their parents-in-law, siblings-in-law, wives, and children. Women, in turn, are primarily responsible for raising and caring for children, from the prenatal period through adulthood, providing guidance and transmitting knowledge and teachings across generations.

For much of the twentieth century, the Javaé and Karajá peoples were regarded as a single Indigenous people, sharing the same cosmology and language. Both groups refer to themselves as Iny, meaning “people.” However, recent studies (Araujo and Nazareno, 2023; Araújo and Nazareno, 2024; Tewaxi, 2019) have demonstrated significant differences between them, some of which may predate colonization. Nevertheless, their similarities led colonizers from the seventeenth and eighteenth centuries onward to classify them as a single Indigenous people. The Javaé inhabit the eastern portion of Bananal Island, whereas the Karajá live in its western portion. Their villages, arranged linearly along the Javaés and Araguaia rivers, comprise sedentary communities whose economies combine agriculture and fishing. Their diet includes fish, turtles, capybaras, and fruits native to the *Cerrado* biome, and they cultivate maize, cassava, beans, and squash. They produce ornaments made from macaw and jabiru feathers and maintain complex ritual systems involving ceremonial moieties, age-based social organization, and institutions such as the Men’s House (Pereira and Nazareno, 2021). They also coexist with other Indigenous peoples, including the Avá-Canoeiro, Tuxá, and Tapirapé.

Their cosmologies express the interconnectedness of animals, plants, humans, and celestial bodies, all understood as possessing intentionality since primordial times. Cultural achievements such as fire, marriage, and reciprocity gave rise to rituals that continue to be practiced today. *Kànỹxiwè* (among the Karajá) and *Tanỹwiwè* (among the Javaé) are regarded as the mythical creator, accompanied by other *aõni*, magical beings that may be benevolent or malevolent. The *Aruanã* (*ijasò*) bring joy, protection, and growth to children (Zanini et al., 2025). Spiritual life, inseparable from material life, constitutes a foundation of identity and collective well-being. Mythological narratives are closely linked to space, the body, and the cosmos. Origin narratives associated with the waters reinforce the centrality of the rivers and lakes of Bananal Island, illustrating how territory functions simultaneously as a physical, symbolic, and identity-forming space for the Iny.

### 2.3 Cultural context of the non-indigenous peoples

As described above, the non-Indigenous group comprised participants who self-identified as White or Brown, were cisgender and heterosexual, and had either incomplete or completed higher education. The group therefore predominantly represented urban, formally educated Brazilian participants. This profile should be considered when interpreting the findings because it does not correspond to the demographic composition of the Brazilian population as a whole. According to the 2022 Brazilian Census, 45.3% of the population self-identified as Brown (*pardo*), 43.5% as White, 10.2% as Black, 0.6% as Indigenous, and 0.4% as Asian (*amarela*) (IBGE, 2023). Brazil is consequently characterized by substantial ethnic-racial, socioeconomic, territorial, and cultural heterogeneity, and the non-Indigenous participants in the present study should not be understood as representative of the broader Brazilian population.

This distinction is particularly relevant to the use of the term *WEIRD* in the present study. Henrich et al. (2010) originally introduced the acronym to characterize populations from Western, Educated, Industrialized, Rich, and Democratic societies and to highlight the disproportionate reliance of psychological research on participants from a relatively narrow set of sociocultural contexts. The term is not used here to characterize Brazil as a WEIRD society. Brazil as a whole cannot be adequately described in these terms, given its marked socioeconomic and cultural heterogeneity and the substantial inequalities that characterize the country. Rather, WEIRD is used here to describe a sampling profile: a group whose sociodemographic characteristics overlap with those of populations that have historically been overrepresented in psychological research.

These inequalities are also relevant because the social characteristics represented in the present non-Indigenous sample should not be interpreted as neutral indicators of Brazilian culture. Rather, they reflect a particular position within a highly unequal society. The concentration of participants with higher educational attainment and urban backgrounds may increase the similarity between this sample and the populations historically identified as WEIRD, even though the broader Brazilian population does not share this profile. Indeed, the Brazilian context is characterized by substantial differences in income and living conditions across social groups and regions (IBGE, 2025). These differences illustrate why a socially and educationally specific convenience sample cannot be assumed to represent Brazilian society as a whole and should be taken as WEIRD-oriented sampling. Therefore, the use of the term WEIRD in this study therefore refers specifically to the characteristics and potential sampling bias of the non-Indigenous participants, rather than to their nationality or to Brazilian society as a whole.

### 2.4 Researcher positionality and reflexivity

The researchers involved in this study have a close and sustained relationship with the topic and with the sociocultural context in which the research was conducted. Two members of the research team have worked for approximately two decades with Indigenous peoples from the Central-West region of Brazil, particularly the Javaé and Karajá peoples, through the Intercultural Education Teacher Education Program at the Federal University of Goiás, which serves students from 32 Indigenous peoples in the region. During this period, they have participated in numerous field-based and collaborative studies addressing Indigenous cosmologies, languages, knowledge systems, health, and food practices. Their work involves regular and reciprocal engagement between the university and Indigenous territories: researchers travel to the territories approximately four times each year, while Indigenous students attend activities at the University twice a year. These encounters constitute sustained spaces of dialogue, collaboration, and knowledge exchange aimed at valuing and strengthening Indigenous knowledge systems.

From a decolonizing perspective, this relationship does not imply the rejection of all Western theories or research traditions, but rather requires that Indigenous concerns, knowledge systems, and ways of understanding the world occupy a central position in the research process (Smith, 2021). Collaborative knowledge production may therefore contribute to ensuring that Indigenous voices are not merely incorporated as sources of data, but actively inform the meanings and interpretations produced through research (Enari et al., 2024). In the present study, particular attention was given to the development of relationships of trust and to maintaining continuous dialogue with Javaé and Karajá participants. As emphasized by Hardin et al. (2023), trust, dialogue, respect for Indigenous ways of producing knowledge, and collaboration are not simply procedural conditions for data collection, but may constitute important dimensions of the research relationship itself.

The other two authors are psychologists whose research focuses on psychological assessment, Positive Psychology, and well-being. Their disciplinary background contributed theoretical and methodological expertise to the study, while also requiring reflexive attention to the assumptions embedded in psychological concepts and frameworks traditionally used to investigate well-being. Accordingly, the research team sought to recognize that its disciplinary and sociocultural positions could influence the questions posed, the analytical categories considered, and the interpretation of participants’ accounts. To reduce the risk of imposing externally defined meanings, the study privileged participants’ own narratives and incorporated dialogical procedures throughout data collection and interpretation. Participants were encouraged to request clarification and negotiate the meanings of the questions and, on several occasions, discussed them among themselves in *Iny Rybè* before formulating their responses in Portuguese. In addition, preliminary findings were subsequently discussed with Javaé and Karajá participants in an operative group, allowing them to recognize, contextualize, question, and further elaborate the researchers’ interpretations. Together, these procedures were intended to strengthen the credibility, reflexivity, and cultural situatedness of the analysis by approaching knowledge production as an intercultural and collaborative process rather than as a unidirectional interpretation of Indigenous experiences by the researchers.

### 2.5 Instruments

Data were collected through three open-ended questions: (1) What makes you happy?; (2) What makes you feel good about yourself and your life?; and (3) What makes your life worth living? In addition, sociodemographic information was collected. For non-Indigenous participants, data included age, gender, and region of residence. For Indigenous participants, information regarding their people and village of residence was collected.

### 2.6 Procedures

Focus Groups (FGs) were conducted to explore participants’ understandings of broad dimensions associated with well-being, through questions addressing happiness, feeling good about oneself and one’s life, and what makes life worth living. These domains were informed by multidimensional psychological approaches to well-being and were used as an initial etic framework rather than assumed to have culturally equivalent meanings across groups. Participants were then asked to respond to the three questions presented above. All focus groups were conducted in Brazilian Portuguese language. A total of six FGs were conducted: three with Indigenous participants (one with Indigenous Karajá men, one with Indigenous Javaé men, and one with Indigenous Javaé women) and three with non-Indigenous participants. The FGs with Indigenous participants lasted an average of 30 min, whereas those with non-Indigenous participants lasted an average of 20 min.

*Indigenous group*. The FGs with Indigenous participants were conducted in the cities of Santa Terezinha, state of Mato Grosso, and Goiânia, state of Goiás, while participants were gathered for activities related to their Intercultural Education degree program at the UFG. The Intercultural Education degree program is offered exclusively to Indigenous peoples from the states of Goiás, Maranhão, Mato Grosso, Minas Gerais, and Tocantins. Its primary objective is to prepare Indigenous teachers to work in Indigenous schools within their communities. The curriculum is organized around contextual themes designed to foster intercultural and interepistemic dialogue between Indigenous and non-Indigenous forms of knowledge. The program follows an alternating pedagogical model, through which students attend classes at the *Takinahakỹ* Indigenous Higher Education Center (NTFSI/UFG) during the months of January, February, July, and August, and remain in their Indigenous territories during April, May, October, and November (UFG, 2019).

In addition to the FGs, an Operative Group was conducted with Indigenous participants to share the findings, promote their joint appraisal and interpretation, and discuss their relevance to participants’ experiences. The results were therefore shared and constructed with participants and collectively discussed. No Operative Group was conduct with non-indigenous group as the authors can represent this population.

Because Portuguese was a second language for the Indigenous participants, particular attention was given to linguistic mediation throughout the data collection process. Participants were encouraged to request clarification whenever the meaning of a question or concept was unclear, and additional explanations were provided when necessary to ensure that the intended meaning of the questions was understood. The interviews were also monitored to verify, as far as possible, the correspondence between the meanings expressed by participants and those recorded by the researchers. This procedure was not based on the assumption of direct equivalence between Portuguese and *Iny Rybè*, but rather on an understanding of translation as a process of linguistic and epistemological mediation in which meanings may require clarification and negotiation. Although conducting interviews in a second language may constitute a potential source of linguistic bias, including possible constraints on explanatory expression and lexical diversity, no systematic comprehension difficulties were observed during the interviews that would indicate such problems as a predominant feature of the data collection process. These procedures were therefore adopted to minimize, while not assuming the complete elimination of, potential effects associated with conducting the interviews in Portuguese.

*Non-Indigenous group*. The FGs conducted with non-Indigenous participants took place in different institutional settings, including classrooms at the Pontifical Catholic University of Goiás, a healthcare unit, and a community center located in Goiânia and Itumbiara. This strategy was adopted to include participants with diverse social and life experiences. Participants were selected through convenience sampling and recruited through direct invitations made by the researchers at the data collection sites. Prior to participation, the objectives of the study, research procedures, and voluntary nature of participation were explained.

### 2.7 Data analysis

The FGs were audio-recorded with participants’ authorization and subsequently transcribed verbatim. The resulting textual corpus was organized into two independent datasets corresponding to Indigenous and non-Indigenous participants. Data analysis followed Charmaz’s (2006) constructivist coding approach and was conducted in two stages: initial coding and focused coding. The entire analytical process was conducted by human researchers, while artificial intelligence-based technologies were used exclusively as exploratory and organizational support tools.

During the initial coding stage, researchers independently conducted line-by-line readings of both corpora and identified preliminary codes. The resulting codes were subsequently discussed collectively in order to achieve consensus and refinement. As an auxiliary resource, word cloud analysis available in the Requalify.ai software (Martins et al., 2024) was used to visualize term frequency within the textual corpus. The word clouds were limited to the 20 most frequent words, with a minimum of two occurrences, providing a preliminary overview of the most salient textual characteristics.

During the focused coding stage, the most recurrent and significant initial codes were synthesized and compared across the Indigenous and non-Indigenous groups. At this stage, Requalify.ai was used to generate graphical representations of semantic networks in order to support the visualization of relationships among codes and emerging themes.

Subsequently, the emerging categories identified in the corpora were analyzed. The development of the analytical categories was carried out by the judges. To enhance reflexivity, methodological transparency, and reduce potential interpretive biases, large language model-based resources were used as exploratory support tools throughout the process. Initially, ChatGPT (GPT-4) was used in a clean session (i.e., without any prior interaction history) to generate preliminary suggestions for thematic grouping. This procedure followed recommendations for the responsible use of artificial intelligence systems in qualitative research, particularly regarding bias mitigation and the preservation of human analytical independence (Yan et al., 2023).

Concurrently, Requalify.ai was used to suggest thematic categories based on semantic patterns identified in the corpus (tag analysis). Suggestions generated by both tools were independently reviewed by the judges and subsequently compared during a joint meeting. All proposed categories were critically evaluated and either refined, combined, or rejected according to the textual corpus, theoretical coherence, and empirical density.

This process resulted in the definition of ten final analytical categories, some shared by both groups and others specific to Indigenous or non-Indigenous participants. The final categories and their respective descriptions were then manually entered into Requalify.ai, and tag analysis was used to identify corpus excerpts associated with each category. The classifications generated by the software were fully reviewed and validated by the judges, who confirmed, adjusted, or corrected the excerpts assigned to each category.

## 3 Results

The analysis of the word clouds generated for the Javaé and Karajá Indigenous groups and the non-Indigenous group highlights the importance of social relationships in shaping perceptions of well-being. The most frequently occurring words were people, family, and friends among non-Indigenous participants, and people, family, village, and community among the Javaé and Karajá Indigenous participants.

However, some significant differences should be noted. Among non-Indigenous participants, the occurrence of words such as money, prosperous, progressing, and successful contrasted with terms such as tranquility, rest, and leisure. This set of words reflects a tension observed in the interviews regarding the perception that well-being is simultaneously composed of personal, professional, and financial success, as well as tranquility, the opportunity to rest, and leisure time. For this group, well-being was also associated with freedom and the ability to consume goods and services considered forms of self-care, such as nail care, hair care, and psychotherapy.

In contrast, among the Javaé and Karajá Indigenous participants, well-being was associated with culture and identity, perceiving oneself as Javaé or Karajá, participating in community life, and fulfilling social roles (e.g., being a teacher, attending university, having students, teaching children, seeking knowledge, and working). Well-being was also linked to participation in everyday activities and rituals, such as fishing, games, and celebrations.

Finally, the analysis of word frequencies revealed marked differences between the groups. Among non-Indigenous participants, only 12 words appeared more than twice in the corpus. Of these, people and family were the most frequent (six occurrences each), followed by friends (four occurrences), peace and healthy (three occurrences each), and connections, happiness, money, tranquility, and rest (two occurrences each).

In contrast, among Indigenous participants, the frequency of occurrence of similar words was substantially higher. The terms happy, people, and family showed the highest frequencies, with 49, 45, and 36 occurrences, respectively. This pattern suggests greater lexical convergence among the Javaé and Karajá Indigenous participants in the way they named and expressed the elements that constitute well-being when compared with non-Indigenous participants, whose references appeared more dispersed throughout the corpus.

Figures 1, 2 present the word clouds generated for Indigenous and non-Indigenous participants.

**Figure 1:**
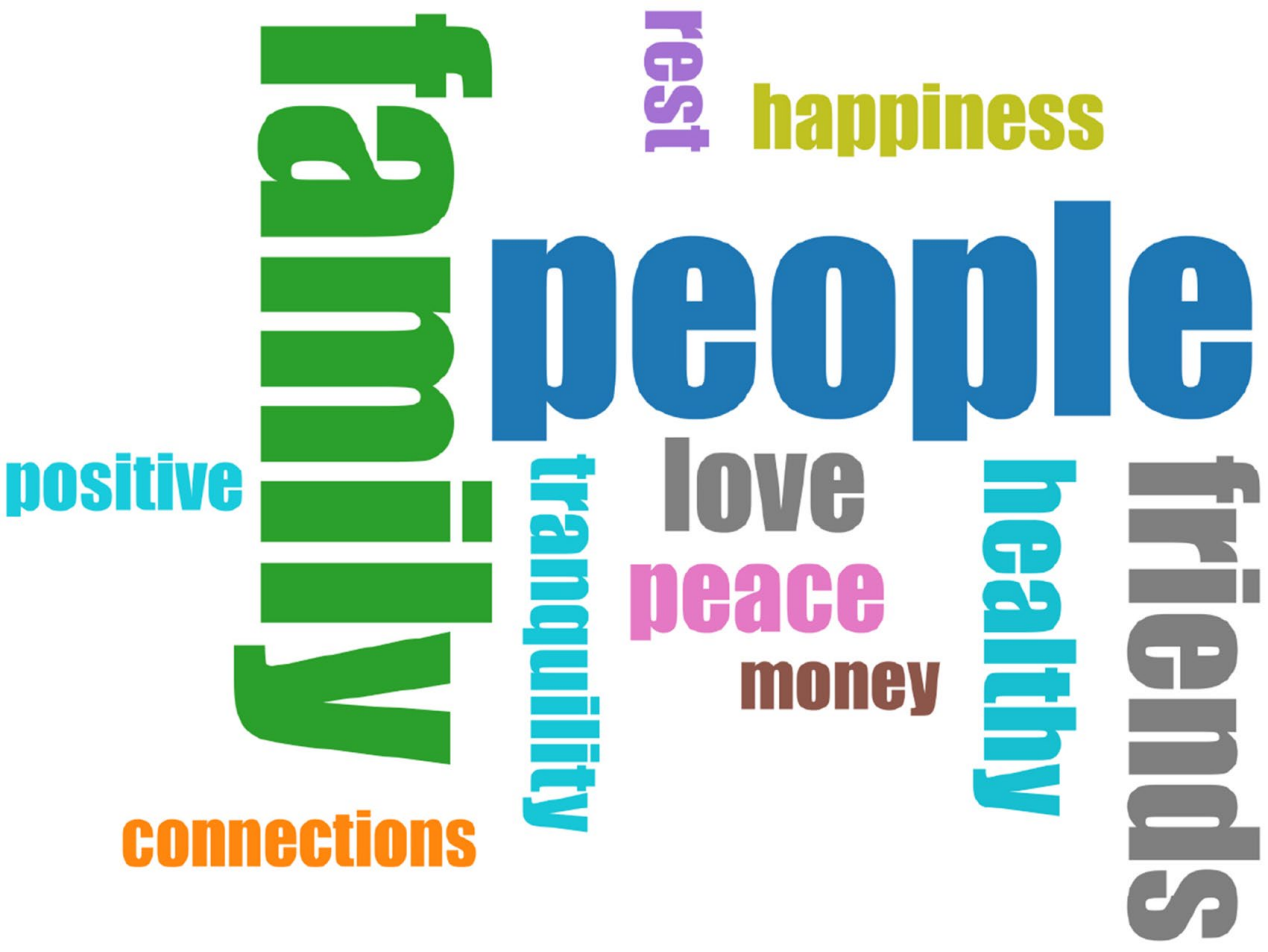
Word cloud of well-being components among non-indigenous participants.

**Figure 2:**
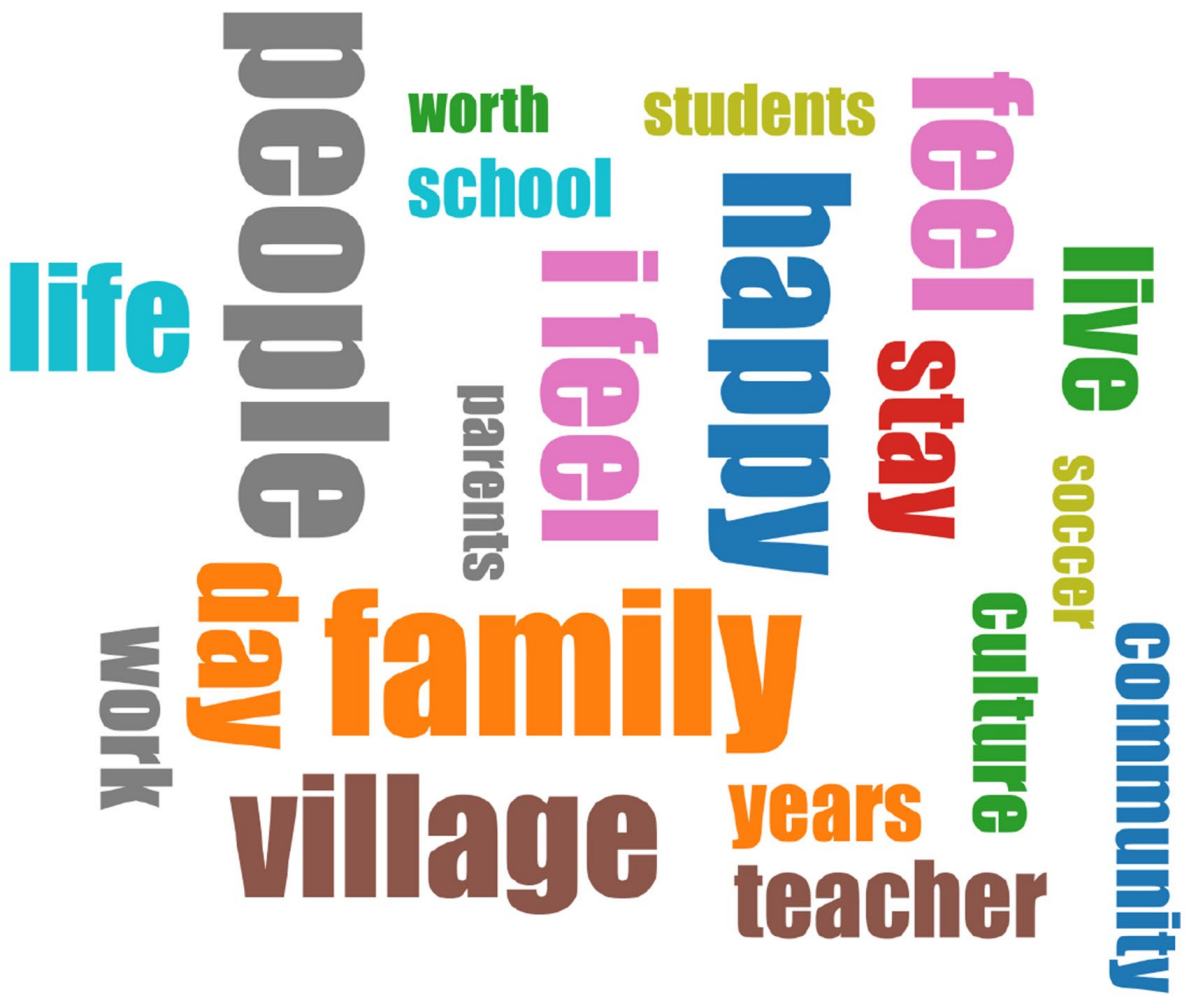
Word cloud of well-being components among Indigenous Javaé and Karajá participants.

According to the interviews, for the non-Indigenous group, well-being and happiness appeared as closely interconnected experiences in participants’ accounts and include or are supported by family. However, well-being depends on the experience of peace and mental health, which includes leisure activities and is influenced by the connections and progress experienced by the individual. In contrast, happiness is associated with tranquility and control and may be supported by physical activity and good nutrition, which is composed of small pleasures. The semantic network of the non-Indigenous participants is presented in Figure 3.

**Figure 3:**
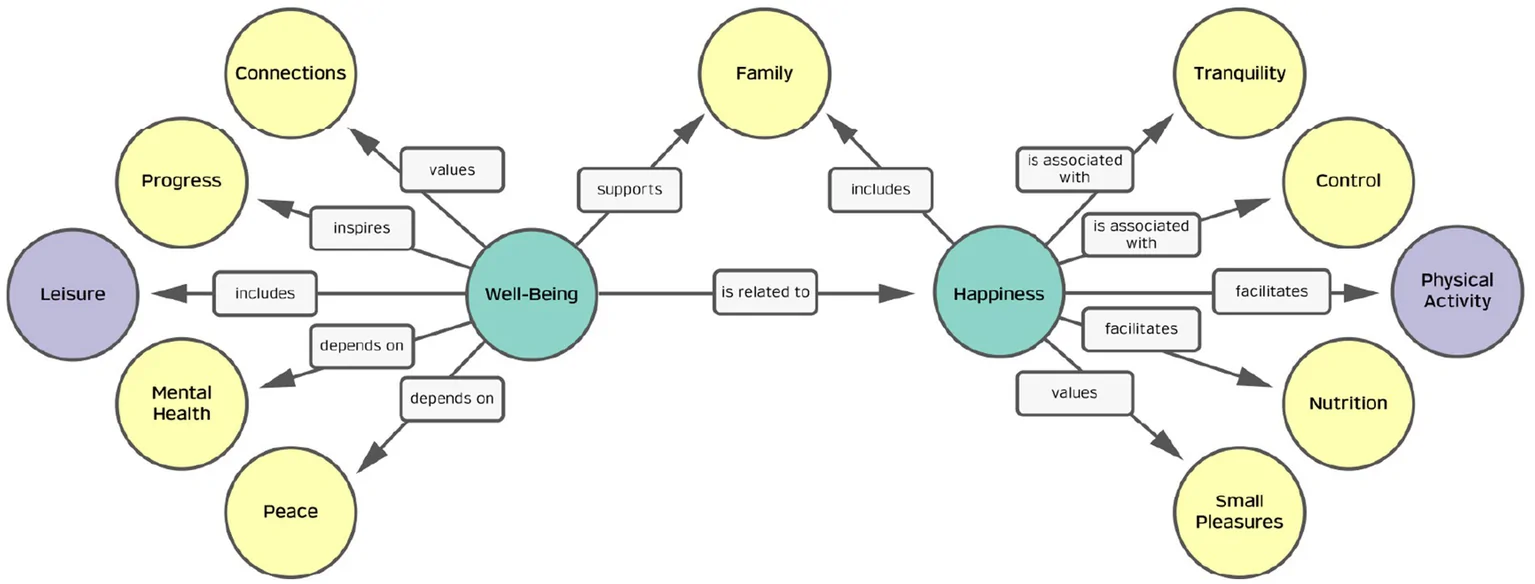
Semantic network of well-being components among non-indigenous participants.

For the Javaé and Karajá Indigenous participants, in contrast, culture is linked to both well-being and spirituality and is grounded in the community. It is reinforced by education, which helps individuals face challenges, promotes satisfaction, and strengthens identity. In this way, culture is connected to well-being, which promotes physical activity, performed primarily in groups, and is linked to happiness. Culture is also connected to well-being when it promotes healthy eating, favoring traditional habits in contrast to processed foods. The semantic network of the Javaé and Karajá Indigenous participants is presented in Figure 4.

**Figure 4:**
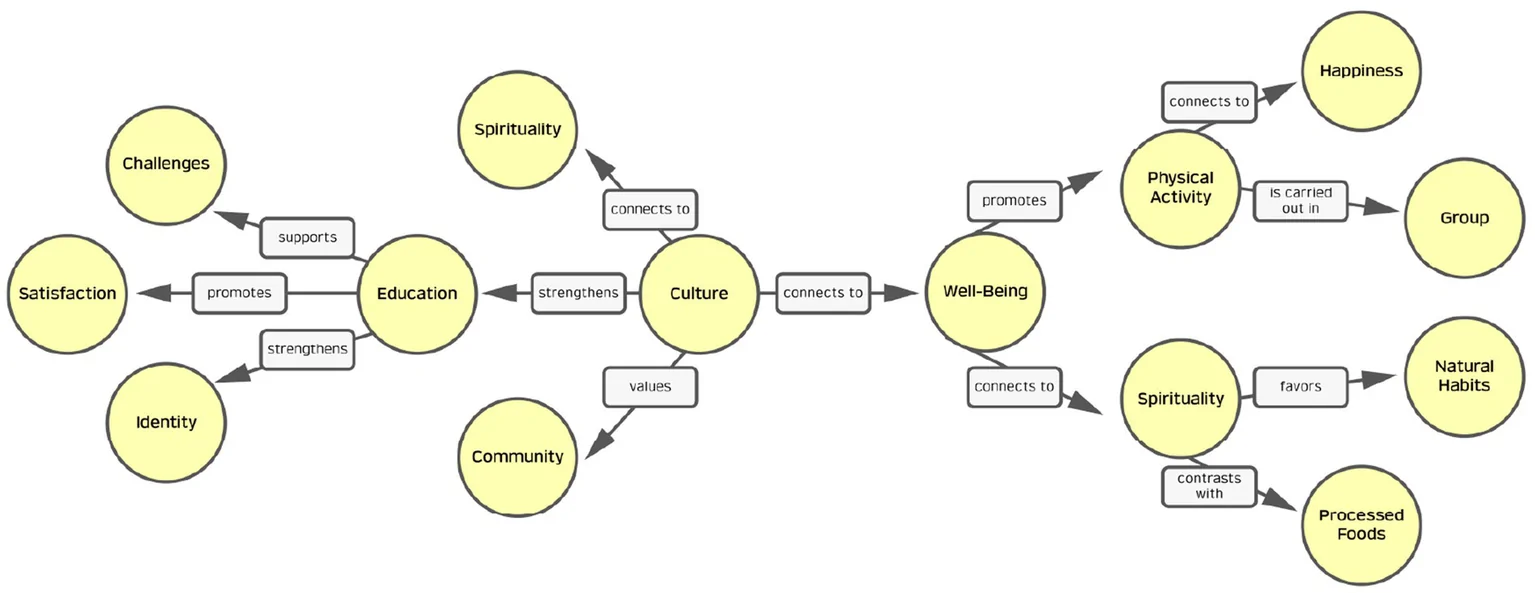
Semantic network of well-being components among indigenous Javaé and Karajá participants.

The analysis of interviews conducted with the Javaé and Karajá Indigenous participants and non-Indigenous participants resulted in the identification of ten categories that express different ways of understanding and experiencing well-being. These categories were grouped into three analytical clusters: (1) categories common to both groups, (2) categories specific to Indigenous participants, and (3) categories more prevalent among non-Indigenous participants. Table 1 presents a synthesis of these categories.

**Table 1:** Description of the categories emerging from the interview corpora.

Categories	Description	F		Examples
		Ind	N-ind	
Family and community relationships	Well-being, happiness, and meaning in life are associated with relationships with family, friends, community, and children.	32	14	*Indigenous*: “being in the village with my family.” (P5) *Non-Indigenous*: “My family, friends, studies, and thinking about the future” (P31).
Meaningful work and teaching	Working as a teacher or being involved in educational activities is a source of satisfaction and purpose.	18	3	*Indigenous*: “working with students the children are my joy.” (P3) *Non-Indigenous*: “My work as a teacher fulfills me. Teaching makes me feel useful” (P33).
Physical and mental health	Healthy eating, physical activity, and mental and emotional health are central factors in feeling well.	6	5	*Indigenous*: “I feel well eating healthy foods and engaging in physical activities” (P1). *Non-Indigenous*: “Being nutritionally healthy, engaging in physical activity, and being mentally healthy” (P32).
Connection with nature and the village	Well-being is linked to experiences within Indigenous territory, including rivers, forests, fishing, open spaces, and traditional practices.	16	0	*Indigenous*: “I feel happy on the banks of the Javaés River. playing soccer and going into the forest” (P11). *Non-Indigenous:* Category: absent.
Indigenous culture, tradition, and belonging	Happiness and meaning in life are strongly connected to culture, traditional celebrations, the transmission of knowledge, and a sense of belonging.	23	0	*Indigenous*: “Participating in traditional celebrations makes me happy” (P8); *Non-Indigenous:* Category: absent.
Self-knowledge and authenticity	Feeling comfortable with one’s own identity, following personal principles, and being true to oneself.	1	9	*Indigenous*: “I feel at peace with myself. I am taking care of my mindset” (P11). *Non-Indigenous*: “Following my principles and being surrounded by people I love” (P34).
Personal growth and realization of aspirations	Studying, accomplishing achievements, and reaching goals are sources of well-being and meaning.	17	4	*Indigenous*: “Being at the university. Everything I ever dreamed of has become reality” (P13). *Non-Indigenous*: “Feeling that I am making progress” (P26).
Spirituality and religiosity	Religion or spirituality as a source of meaning and guidance in life.	4	1	*Indigenous*: “Seeking God, because without Him we are lost in the world” (P11). *Non-Indigenous:* “I have faith, but I do not follow any religion” (P30).
Overcoming personal challenges	Facing and overcoming addictions, emotional difficulties, or adverse circumstances as a way of restoring well-being.	6	2	*Indigenous*: “Alcohol interfered with my life. now I am happy with my family” (P2). *Non-Indigenous:* “A sense of control over my life” (P31).
Immediate pleasures and consumption	Activities associated with immediate gratification (e.g., sex, alcohol, money, appearance, and consumption) as sources of happiness or well-being.	2	9	*Indigenous: “*It is like this, (drinking) socially. Not getting drunk, right?” (P9). *Non-Indigenous*: “Alcohol, games, sex, conversations” (P24).

F, Frequency; Ind, Javaé and Karajá Indigenous participants; N-Ind, Non-Indigenous participants; P, Participant.

Among the categories common to both groups and showing comparable frequencies, the following stood out: Family and community relationships, strongly associated with the construction of social bonds, mutual support, and a sense of belonging; Meaningful work and teaching, linked to professional fulfillment and the value attributed to teaching and the exchange of knowledge; Physical and mental health, understood as a prerequisite for well-being and autonomy; and Personal growth and realization of aspirations, associated with achieving goals and personal accomplishments, such as obtaining a university degree or achieving financial stability.

However, it is important to note that these convergences point to dimensions of well-being that, although shared, are mobilized through distinct cultural repertoires. In the case of Family and community relationships, the Javaé and Karajá participants emphasized the centrality of the village and an extended relational network, whereas non-Indigenous participants tended to locate well-being primarily within the nuclear family and close interpersonal relationships. Regarding Meaningful work and teaching, although teaching emerged in both groups, among Indigenous participants it was associated with returning to the village and contributing to the collective, whereas among non-Indigenous participants it was more closely linked to individual trajectories and recognition.

With respect to Physical and mental health, Indigenous participants’ accounts suggested an understanding connected to village practices and traditions, whereas non-Indigenous participants tended to describe it in more individualized terms. Finally, regarding Personal growth and realization of aspirations, entry into higher education emerged among Indigenous participants as part of a collective project of returning knowledge to the community. Among non-Indigenous participants, greater emphasis was placed on career advancement and personal achievement.

In addition, Spirituality and religiosity were present in both groups, but appeared with greater frequency and symbolic significance among Indigenous participants, for whom the relationship with the sacred plays an important role in guiding life and coping with adversity. The category Overcoming personal challenges, although identified in both contexts, was substantially more frequent among Indigenous participants, reflecting processes of resilience in response to historical suffering, discrimination, and the challenges encountered throughout their life and educational trajectories. In contrast, the category Self-knowledge and authenticity was more prevalent among non-Indigenous participants and was associated with consistency between personal values and identity.

Among the categories specific to Indigenous participants, two stood out: Connection with nature and the village, as a direct source of sensory and spiritual well-being, evoking the river, the forest, and ritual practices; and Indigenous culture, tradition, and belonging, encompassing the use of the mother tongue, participation in traditional celebrations, and pride in Indigenous identity.

These categories express ways of experiencing well-being deeply rooted in collectivity, territory, and ancestry, highlighting relational and culturally situated meanings of living well that transcends the individualized logic predominant in Western models. Among non-Indigenous participants, the category Immediate pleasures and consumption stood out, encompassing references to consumption, aesthetics, and sensory experiences associated with leisure and self-fulfillment.

Finally, an operative group was conducted with the Indigenous Javaé and Karajá participants to share the findings, promote their joint appraisal and interpretation, and discuss their relevance to participants’ experiences. The meeting was not conceived merely as a validation stage, but also as a space for collaborative interpretation of the findings. Participants reported recognizing themselves and feeling represented in the overall set of results. They expressed satisfaction when seeing their responses represented in the word clouds, recognizing elements of the group’s accounts, and in the semantic networks, in which they identified the explanatory pathway they had used to understand well-being from their own perspectives.

The dialogue also enabled the identification and contextualization of aspects that could not be fully understood from the originally analyzed data alone. For example, Javaé women highlighted the absence of references to sports activities in their accounts and explained that this absence was related to the fact that, in their culture, women do not participate in the “games” or recreational activities mentioned by the men. This contribution provided contextualization for a difference observed in the representation of the data and was incorporated into the interpretation of the findings. Another relevant aspect emerged from the discussion with Javaé and Karajá men, who emphasized the impact of alcohol consumption on community relationships, health, and well-being. Although this aspect was already present in the representation of their accounts, participants highlighted its contemporary relevance for understanding the well-being of both Indigenous peoples. Thus, the operative group contributed not only to confirming the relevance of the findings, but also to contextualizing, deepening, and refining their interpretation based on the knowledge and lived experiences of the participants themselves.

## 4 Discussion

This study aimed to analyze perceptions of well-being, happiness, and meaning in life among Indigenous participants from the Iny, Javaé and Karajá peoples, and non-Indigenous participants, seeking to identify both shared aspects and group-specific aspects and discussing them into etic and emic perspective. Examining these elements makes it possible to understand well-being as a plural and situated experience shaped by distinct value systems, modes of subjectivation, and ontological frameworks (Helfrich, 1999; Sollis et al., 2024).

The findings should be interpreted in relation to the multidimensional framework adopted as the initial etic point of departure for the study. The three guiding questions addressed experiences associated with happiness, feeling well about oneself and one’s life, and what makes life worth living. In participants’ accounts, these domains were not articulated as sharply separated experiences. Rather, they were frequently interconnected within broader descriptions of what contributes to living well. This pattern does not imply conceptual equivalence between happiness, meaning in life, and well-being. Instead, it indicates that, within the semantic organization of participants’ accounts, the experiences elicited by the three questions were closely related. The analysis therefore focuses primarily on how the broad dimensions mobilized by the initial well-being framework were interpreted, connected, and culturally situated by participants in each group.

Overall, the findings suggest that although common elements exist in the ways participants describe well-being, these elements are organized and experienced through profoundly different cultural frameworks, pointing to the emic aspects that sould be taken into account when studying different ethnic groups. The analyses highlight the centrality of family and community relationships for both groups, corroborating findings from the literature regarding the role of perceived family support in promoting well-being among adults (An et al., 2024). However, this apparent convergence does not imply equivalence in meaning or cultural significance, as family and community relationships are situated differently within the cultural contexts examined.

Among the Javaé and Karajá peoples, family, community, village, and identity constitute an expanded relational system structured around principles such as reciprocity, generosity, and contribution to the collective. According to Rodrigues (2023), the family constitutes the fundamental unit of Iny societies, integrating physical, ceremonial, and spiritual dimensions. The care of mythical beings such as the *Aruanã* through rituals and offerings extends the notion of kinship beyond the human realm, articulating family, community, and cosmology within a shared logic of belonging. In this sense, well-being emerges as a collective experience sustained by the continuity of relationships among humans, spirits, territory, and ancestry.

The centrality of ritual practices further reinforces this relational understanding of well-being. Collective preparations for the arrival of the *Aruanã* during the *Hetohokỹ* ritual not only reaffirm community ties but also renew collective identity and shared responsibility for maintaining social and cosmological balance. Thus, the sense of well-being described by Indigenous participants cannot be separated from ritual experience, community reciprocity, and the maintenance of cosmological order (Rodrigues, 2023).

Among non-Indigenous participants, although family relationships also emerged as relevant to well-being, they tended to be understood through a framework more closely restricted to the nuclear family and intimate interpersonal bonds, grounded in conceptions of individuality and autonomy (Gardiner et al., 2020). Thus, family and community relationships constitute a point of convergence between the groups, but again they are organized according to distinct conceptions of the self, collectivity, and belonging, in an emic perspective (Helfrich, 1999).

Importantly, the collaborative discussion of the findings with the Javaé and Karajá participants further demonstrated the relevance of incorporating Indigenous perspectives into the interpretation of the data. Participants not only recognized themselves in the word clouds and semantic networks but also contributed contextual interpretations that refined the researchers’ understanding of the findings. For example, Javaé women explained that the absence of references to sports activities in their accounts was related to gendered cultural practices, as women do not participate in the “games” mentioned by the men. Likewise, Javaé and Karajá men emphasized the current impact of alcohol consumption on community relationships, health, and well-being, highlighting its contemporary relevance beyond its frequency in the original accounts. These contributions illustrate that collaboration with Indigenous participants can move beyond the confirmation of predefined interpretations, allowing culturally situated knowledge to qualify and deepen the analysis. In this sense, recognizing participants as contributors to the interpretation of their own experiences is consistent with approaches that challenge the universalization of Western psychological categories and emphasize the importance of culturally grounded forms of knowledge production (Christopher and Hickinbottom, 2008; Prilleltensky, 2013; Lomas et al., 2021; Lomas, 2022).

This collaborative process should also be understood in light of the challenges involved in intercultural translation. We did not assume that the methodological procedures employed could provide complete or transparent access to an Indigenous perspective. Rather, following de Castro’s (2005) notion of equivocal translation, we understand translation as an operation that brings perspectives into relation without presuming that they are saying the same thing or that their differences can be fully resolved. In the present study, the Indigenous participants first sought clarification of the questions and concepts under discussion, raised additional questions when necessary, and subsequently discussed the issues among themselves, including in their own language, Iny Rybè, before presenting their interpretations to the researchers. This process can be understood as a form of dialogical multiplication, in which intercultural encounters do not eliminate differences between perspectives but may expand the possibilities of dialogue by bringing otherwise marginalized epistemic positions into the production and interpretation of knowledge (Guimarães, 2017). In this sense, intercultural translation should not be understood as a means of achieving a complete or definitive emic representation. Rather, it constitutes a dialogical process through which meanings can be negotiated, differences made visible, and forms of knowledge historically marginalized by Eurocentric epistemologies incorporated into the research process (Nazareno and Meneses, 2023). The interpretations presented in this study should therefore be understood not as a transparent representation of Indigenous worlds, but as situated understandings produced through an intercultural and interepistemic encounter.

This perspective also requires acknowledging the linguistic and epistemological mediations involved in the research process itself. Indeed, such mediations involve important challenges. In this sense, the present study can be understood as involving a critical transposition of a psychological framework of well-being developed predominantly within Western scholarship. The framework informed the broad domains addressed by the guiding questions, but their meanings were not assumed to be universal or directly transferable across cultural contexts. Rather, the analysis examined how these domains were interpreted, re-signified, and related within the participants’ situated systems of meaning. Rather, the research is also grounded in a perspective that problematizes the universalization of categories and frameworks produced within hegemonic epistemological contexts and seeks to understand how such frameworks are re-signified and negotiated within situated contexts. Accordingly, the relationship between Portuguese and *Iny Rybè* should not be understood exclusively as an operation of translating content from one language into another. Instead, the research involved a process of mediation and negotiation of meanings in which different ways of expressing and producing knowledge were taken into consideration. We sought to recognize the historical asymmetries involved in knowledge production while simultaneously considering the participants’ own forms of knowledge and meaning-making as important elements of the analysis. In this sense, language was approached not merely as a medium of communication, but as an epistemological space in which categories mobilized by the research could be negotiated.

This ontological difference is also reflected in the ways other components of well-being are articulated. Categories such as Physical and mental health, Meaningful work and teaching, and Personal growth and realization of aspirations emerged as relevant in both groups, yet once again assumed different meanings. The findings indicate a holistic understanding of health that integrates physical, emotional, and psychological dimensions. However, among Indigenous peoples, this understanding is deeply rooted in ancestral practices, such as traditional foodways, fishing, everyday physical activity, and rituals, whereas among non-Indigenous participants, health tends to be associated with individualized practices and the contemporary wellness paradigm, often marked by the commodification of well-being (Reppold et al., 2019).

Similarly, Meaningful work and teaching reveals itself as a point of convergence only at a superficial level. Among the Javaé and Karajá peoples, work is intrinsically connected to collectivity, cultural reproduction, and the maintenance of social organization, particularly within the context of the matrilineal structure. Productive activities such as farming, fishing, and hunting ensure not only material subsistence but also the continuity of the social and symbolic relationships that sustain collective life (Rodrigues, 2008). Among non-Indigenous participants, in contrast, meaningful work was more frequently associated with individual trajectories, social recognition, and personal success, reflecting meritocratic and individualizing values.

This distinction becomes even more apparent in the analysis of the category Personal growth and realization of aspirations. For non-Indigenous participants, this category was strongly linked to academic, professional, or athletic achievements, oriented toward ideals of success and accomplishment (Kovich et al., 2023). Among Indigenous participants, the realization of aspirations assumed a collective and political character, being associated with occupying spaces that have historically been denied to Indigenous peoples, such as universities. In this context, gaining access to and remaining within these spaces reaffirms cultural identity, generates personal satisfaction, and functions as a strategy for confronting structural inequalities. However, this data should be taken with caution. It is worth noting that all indigenous participants were students of the Intercultural Degree program at UFG, selected by convenience sampling, which may affect the results. Despite this, it should be emphasized that they were also teachers in their communities at schools focused on indigenous school education, thereby linked to cultural preservation.

The categories particularly salient among Indigenous participants, such as Connection with nature and the village and Indigenous culture, tradition, and belonging, reveal a relational conception of well-being that, while grounded in the participants’ own sociocultural context, presents points of dialogue with broader Latin American perspectives associated with *Buen Vivir*. Territory, rivers, forests, rituals, and the mother tongue are not secondary elements, but constitutive dimensions of the experience of existing in the world. For the Javaé and Karajá peoples, there is no separation between culture and nature; life is shaped by ongoing relationships among humans, animals, spirits, and mythical beings, regardless of their ontological condition (Tewaxi, 2019). This perspective contrasts with the anthropocentric foundations of Western Psychology, revealing an implicit critique of the separation between the self and nature.

The notion of well-being as a process is also evident in the category Overcoming personal challenges, which was particularly prominent in Indigenous narratives. Confronting adversities such as racism, school dropout, poverty, and identity conflicts frames well-being not as a static state to be attained, but as an ongoing process of resistance, adaptation, and self-reconstruction.

Spirituality, although present in both groups, acquires greater symbolic significance among Indigenous participants, as it is articulated through collective cosmologies and spiritual strength as an ethical and practical foundation of life. Among non-Indigenous participants, spirituality tends to appear in a more individualized form, often detached from a relational ontology. In contrast, categories such as immediate pleasures, hedonism, and consumption emerged more frequently among non-Indigenous participants, reflecting contemporary values associated with sensory enjoyment, aesthetics, and the valorization of consumption as a source of personal satisfaction (Reppold et al., 2019), elements that were absent from Indigenous narratives.

Finally, differences in the frequency of terms related to meaningful work and the realization of aspirations should not be interpreted simply as the absence of particular values among Indigenous peoples, but rather understood in light of their own moral and ethical categories. Among the Iny, Javaé and Karajá peoples, individuals are considered good or morally upright when their actions express generosity, reciprocity, and commitment to the collective. Social and symbolic organization reinforces this relational logic, in which women and men perform complementary and essential roles in the production and continuity of social life, without hierarchical distinctions between genders (Rodrigues, 2008).

## 5 Final considerations

In summary, the findings reinforce the limitations of uncritically approaching well-being as a universally equivalent construct through models and parameters developed predominantly from WEIRD populations. The analysis of convergences and divergences in the meanings associated with dimensions of well-being across Indigenous and non-Indigenous participants reinforces the importance of intercultural and decolonial approaches to Psychology committed to epistemic justice and to the recognition of the ontological, cultural, and political plurality of human experiences of Well-being.

By presenting empirical evidence based on the accounts of members of two distinct Indigenous peoples, this study contributes to broadening and challenging hegemonic models of well-being, bringing Indigenous worldviews into dialogue with the field and highlighting the relevance of collective, territorial, and cultural dimensions for understanding this phenomenon.

More broadly, this study contributes to current efforts to diversify psychological science by highlighting the limitations of interpretations that do not adequately consider culturally situated, emic perspectives of well-being; and that Indigenous knowledge systems are not merely cultural variations of supposedly universal theories but offer conceptual resources capable of expanding contemporary understandings of well-being. We therefore hope the manuscript will stimulate dialogue between Cultural Psychology, Positive Psychology, and Indigenous psychologies, fostering more inclusive and globally representative theories of human flourishing.

Nevertheless, the results should be interpreted in light of their limitations. Access to only two Indigenous peoples in a country characterized by extensive Indigenous diversity means that the findings represent a situated approximation of Indigenous worldviews regarding well-being, without any claim to encompass their full scope or internal diversity. Therefore, the findings should not be generalized to all Indigenous peoples in Brazil. Another limitation refers to the gender representation of the sample. As noted we used a convenience sample of Intercultural students of UFG that include both men and women for Javaé people but only men for Karajá group. As pointed before in the matrilineal social structures of the Karajá and Javaé, women play a dominant role in the intergenerational transmission of knowledge, so the absence of Karajá women can result in an incomplete emic perspective of the Karajá group.

Finally, some caution is warranted regarding the use of *Buen Vivir* as an interpretive framework. In this study, *Buen Vivir* was not assumed to be equivalent to an Iny conception of well-being, nor was it used as an overarching category to represent Indigenous ways of living. Rather, the analysis privileged the meanings articulated by Javaé and Karajá participants, and *Buen Vivir* was subsequently mobilized as a framework for dialogue with the relational and collective dimensions that emerged from the findings. Future research exploring concepts expressed specifically in Iny Rybè may further enrich the understanding of well-being from Javaé and Karajá perspectives.

Future studies could extend this investigation by examining conceptions of well-being across a broader and more diverse range of Indigenous peoples, both within Brazil and in other sociocultural and territorial contexts. Such comparative research may help identify possible convergences while preserving the culturally and epistemologically situated meanings through which each people understands and experiences well-being, thereby advancing the dialogue between etic and emic perspectives and contributing to the development of more culturally sensitive and inclusive theoretical models and assessment instruments.

## Data Availability

The data supporting the conclusions of this article may be made available by the authors upon reasonable request, subject to ethical, confidentiality, and participant privacy considerations.

## References

[ref1] Acosta A. (2016). O bem viver: Uma oportunidade para imaginar outros mundos (T. Breda, Trad.). São Paulo, Brazil: Autonomia Literária.

[ref2] An J. Zhu X. Shi Z. An J. (2024). A serial mediating effect of perceived family support on psychological well-being. BMC Public Health 24:940. doi: 10.1186/s12889-024-18476-z, 38566105 PMC10986067

[ref3] Araujo O. C. G. Nazareno E. (2023). “Isolamento voluntário” e protagonismo do povo Javaé, da Ilha do Bananal, atual estado do Tocantins-Brasil (1896–1923). Hist. Rev. 27, 26–48. doi: 10.22456/1982-6524.139482

[ref4] Araújo O. C. G. Nazareno E. (2024). Povos indígenas no Vale do Araguaia, Brasil: Evangelização protestante e projeto de civilização na Primeira República (1897–1930). Espaço Ameríndio 18, 52–69.

[ref5] Charmaz K. (2006). Constructing Grounded Theory: A Practical guide through Qualitative Analysis. London, UK: Sage Publications.

[ref6] Christopher J. C. Hickinbottom S. (2008). Positive psychology, ethnocentrism, and the disguised ideology of individualism. Theory Psychol. 18, 563–589. doi: 10.1177/0959354308093396

[ref7] de Castro E. V. (2005). “Equívocos da identidade,” in O que é memória social? eds. Gondar J. Dodebei V. (Rio de Janeiro, Brazil: Contra Capa), 145–160.

[ref8] Deci E. L. Ryan R. M. (2002). Handbook of Self-Determination Research. Rochester, NY, United States: University of Rochester Press.

[ref9] Diener E. (1984). Subjective well-being. Psychol. Bull. 95, 542–575. doi: 10.1037/0033-2909.95.3.542, 6399758

[ref10] Enari D. Matapo J. Ualesi Y. Cammock R. Port H. Boon J. . (2024). Indigenising research: Moanaroa a philosophy for practice. Educ. Philos. Theory 56, 1044–1053. doi: 10.1080/00131857.2024.2323565

[ref11] Gardiner G. Lee D. Baranski E. Funder D. C. Members of the International Situations Project (2020). Happiness around the world: a combined etic–emic approach across 63 countries. PLoS One 15:e0242718. doi: 10.1371/journal.pone.0242718, 33296388 PMC7725360

[ref12] Guimarães D. S. (2017). Multiplicação dialógica: Ensaios de psicologia cultural [Tese de livre-docência, Universidade de São Paulo]. São Paulo, Brazil: Instituto de Psicologia, Universidade de São Paulo.

[ref13] Hardin J. Enari D. Phillips T. Aiavao T. Boodoosingh R. Fulu Aiolupotea S. A. M. . (2023). Developing trust in collaborative research: utilizing indigenous Pacific methodologies to create dialogue within research teams. Asia Pacific J. Public Health 35, 529–531. doi: 10.1177/10105395231204809, 37864308

[ref14] Helfrich H. (1999). Beyond the dilemma of cross-cultural psychology: resolving the tension between etic and emic approaches. Cult. Psychol. 5, 131–153. doi: 10.1177/1354067X9952002

[ref15] Henrich J. Heine S. J. Norenzayan A. (2010). The weirdest people in the world? Behav. Brain Sci. 33, 61–83. doi: 10.1017/S0140525X0999152X, 20550733

[ref16] IBGE (2023). Censo Demográfico 2022: Identificação étnico-racial da população, por sexo e idade: Resultados do universo. IBGE. Rio de Janeiro, Brazil: IBGE.

[ref17] IBGE (2025). Síntese de indicadores sociais: Uma análise das condições de vida da população brasileira: 2025. Rio de Janeiro, Brazil: IBGE.

[ref18] Kovich M. K. Simpson V. L. Foli K. J. (2023). Application of the PERMA model of well-being in undergraduate students. Int. J. Community Well-Being 6, 1–20. doi: 10.1007/s42413-022-00184-4, 36320595 PMC9607835

[ref9001] Lomas T. (2022). The atlas of happiness: The global secrets of how to be happy. Bluebird.

[ref19] Lomas T. Waters L. Williams P. Oades L. G. Kern M. L. (2021). Third wave positive psychology: broadening towards complexity. J. Posit. Psychol. 16, 660–674. doi: 10.1080/17439760.2020.1805501

[ref20] Martins L. F. Souza F. R. Freitas J. M. (2024) Requalify.Ai (version 0.1) [Software]. Available online at: https://requalify.ai (Accessed September 16, 2026)

[ref21] Nazareno E. Meneses M. P. (2023). Educação intercultural indígena na Universidade Federal de Goiás: Escolas indígenas e o papel dos temas contextuais nas escolas indígenas. Rev. Latinoam. Estud. Educ. 53, 125–155. doi: 10.48102/rlee.2023.53.3.584

[ref22] Pereira T. M. G. Nazareno E. (2021). “Tradicionalizando o Ixỹju: A alimentação Javaé na contemporaneidade,” in Violência, gênero e populações vulneráveis, eds. Grubits S. de Oliveira E. Vera Noriega J. Á., vol. 1 (Campo Grande, MS, Brazil: Editora UCDB), 115–136.

[ref23] Pincus J. D. (2024). Well-being as need fulfillment: implications for theory, methods, and practice. Integr. Psychol. Behav. Sci. 58, 1541–1579. doi: 10.1007/s12124-023-09758-z, 37022593 PMC10078020

[ref24] Prilleltensky I. (2013). Wellness without fairness: the missing link in psychology. S. Afr. J. Psychol. 43, 147–155. doi: 10.1177/0081246313484238

[ref25] Reppold C. T. Zanini D. S. Campos D. C. Faria M. R. G. V. Tocchetto B. S. (2019). Felicidade como produto: Um olhar crítico sobre a ciência da Psicologia Positiva. Aval. Psicol. 18, 333–342. doi: 10.15689/ap.2019.1804.18777.01

[ref26] Rodrigues P. M. (2008). A caminhada de Tanỹxiwè: Uma teoria Javaé da história (Tese de doutorado). Chicago, IL, United States: University of Chicago.

[ref27] Rodrigues P. d. M. (2023). “Brincadeiras do corpo: Gênero e historicidade Javaé em diálogo com a perspectiva de Rita Segato,” in Encontro com Rita Segato por sua linhagem: Memórias e memorial, ed. de Almeida T. M. C. (Brasília, DF, Brazil: Editora Universidade de Brasília), 172–185.

[ref28] Ryff C. D. (1989). Happiness is everything, or is it? Explorations on the meaning of psychological well-being. J. Pers. Soc. Psychol. 57, 1069–1081. doi: 10.1037/0022-3514.57.6.1069

[ref29] Ryff C. D. (2022). Positive psychology: looking back and looking forward. Front. Psychol. 13:840062. doi: 10.3389/fpsyg.2022.840062, 35369156 PMC8967995

[ref30] Seligman M. E. P. (2011). Florescer: Uma nova compreensão visionária da felicidade e do bem-estar. New York, NY, United States: Free Press.

[ref31] Seligman M. E. P. (2018). PERMA and the building blocks of well-being. J. Posit. Psychol. 13, 333–335. doi: 10.1080/17439760.2018.1437466

[ref32] Smith L. T. (2021). Decolonizing Methodologies: Research and Indigenous Peoples. 3rd Edn London, UK: Zed Books.

[ref33] Sollis K. Biddle N. Maulana H. Yap M. Campbell P. (2024). Measuring wellbeing across culture and context: are we getting it right? Evaluating the variation in wellbeing conceptualisations throughout the world. Soc. Indic. Res. 174, 123–155. doi: 10.1007/s11205-024-03382-z

[ref34] Tewaxi R. J. (2019). Nas águas do rio Javaés: Histórias, cosmologia e meio ambiente (Dissertação de mestrado). Universidade Federal do Tocantins.

[ref35] UFG (2019) Projeto pedagógico do curso de Educação Intercultural. Núcleo Takinahakỹ de Formação Superior Indígena. Available online at: https://intercultural.letras.ufg.br/ (Accessed September 16, 2026)

[ref36] Yan L. Echeverria V. Fernandez Nieto G. Jin Y. Swiecki Z. Zhao L. . (2023). Human–AI collaboration in thematic analysis using ChatGPT: a user study and design recommendations. arXiv. [Preprint] doi: 10.48550/arXiv.2311.039999

[ref37] Zanini D. S. Fernandes I. A. Nazareno E. Mendonça T. M. G. P. (2025). Colonialidades do bem-estar: Perspectivas indígenas Karajá e Javaé e não indígenas. Psicologia: Ciência e Profissão 45:e299202. doi: 10.1590/1982-3703003299202, 41099703

[ref38] Zhang W. Balloo K. Hosein A. Medland E. (2024). A scoping review of well-being measures: conceptualisation and scales for overall well-being. BMC Psychol. 12:585. doi: 10.1186/s40359-024-02074-0, 39443963 PMC11515516

